# Inhibition of repulsive guidance molecule-a protects dopaminergic neurons in a mouse model of Parkinson’s disease

**DOI:** 10.1038/s41419-021-03469-2

**Published:** 2021-02-15

**Authors:** Wakana Oda, Yuki Fujita, Kousuke Baba, Hideki Mochizuki, Hitoshi Niwa, Toshihide Yamashita

**Affiliations:** 1 Department of Molecular Neuroscience, Graduate School of Medicine, Osaka University, 2-2 Yamadaoka, Suita, Osaka 565-0871 Japan; 2 Department of Dental Anesthesiology, Graduate School of Dentistry, Osaka University, 1-8 Yamada-Oka, Suita, Osaka 565-0871 Japan; 3 World Premier International, Immunology Frontier Research Center, Osaka University, 3-1 Yamadaoka, Suita, Osaka 565-0871 Japan; 4 Department of Neurology, Graduate School of Medicine, Osaka University, 2-2 Yamadaoka, Suita, Osaka 565-0871 Japan; 5 Graduate School of Frontier Biosciences, Osaka University, 2-2 Yamadaoka, Suita, Osaka 565-0871 Japan; 6 Department of Neuro-Medical Science, Graduate School of Medicine, Osaka University, 2-2 Yamadaoka, Suita, Osaka 565-0871 Japan

**Keywords:** Cell death in the nervous system, Parkinson's disease

## Abstract

Repulsive guidance molecule-a (RGMa), a glycosylphosphatidylinositol-anchored membrane protein, has diverse functions in axon guidance, cell patterning, and cell survival. Inhibition of RGMa attenuates pathological dysfunction in animal models of central nervous system (CNS) diseases including spinal cord injury, multiple sclerosis, and neuromyelitis optica. Here, we examined whether antibody-based inhibition of RGMa had therapeutic effects in a mouse model of Parkinson’s disease (PD). We treated mice with 1-methyl-4-phenyl-1,2,3,6-tetrahydropyridine (MPTP) and found increased RGMa expression in the substantia nigra (SN). Intraventricular, as well as intravenous, administration of anti-RGMa antibodies reduced the loss of tyrosine hydroxylase (TH)-positive neurons and accumulation of Iba1-positive microglia/macrophages in the SN of MPTP-treated mice. Selective expression of RGMa in TH-positive neurons in the SN-induced neuronal loss/degeneration and inflammation, resulting in a progressive movement disorder. The pathogenic effects of RGMa overexpression were attenuated by treatment with minocycline, which inhibits microglia and macrophage activation. Increased RGMa expression upregulated pro-inflammatory cytokine expression in microglia. Our observations suggest that the upregulation of RGMa is associated with the PD pathology; furthermore, inhibitory RGMa antibodies are a potential therapeutic option.

## Introduction

Parkinson’s disease (PD) is a neurodegenerative disorder that presents with a variety of motor and non-motor symptoms^1^. The progressive decline in body movements in PD results from reduced dopamine production in the substantia nigra (SN). Repulsive guidance molecule-a (RGMa) expression is increased in the SN in PD^2^. Accumulating studies in neurological diseases spinal cord injury (SCI) and multiple sclerosis (MS) demonstrate that increased expression of RGMa inhibits axon regeneration and functional recovery in the injured CNS^3–6^. RGMa can be an inhibitory factor of neuroplasticity in neurodegenerative diseases.

RGM is a cell membrane-associated glycosylphosphatidylinositol-anchored glycoprotein that was originally identified as an axon repellent in the chick retinotectal system^7–9^. RGMa has a diverse physiological function, such as to inhibit axon growth^10–12^. In adult rats with SCI, RGMa express around the lesion site both in neurons and non-neuronal cells, such as oligodendrocytes and microglia. Loss of function by local treatment with RGMa-neutralizing antibodies significantly promotes axon regeneration after SCI^10^. The antibody-based inhibition of RGMa facilitates axonal growth, ameliorates inflammation, and increases remyelination in models of MS^13,14^. RGMa overexpression in the SN induces loss of tyrosine hydroxylase (TH)-positive neurons and glial activation^15^. We thus hypothesized that RGMa inhibition has therapeutic effects in PD. In this study, we examined whether intraventricular treatment with polyclonal antibodies against RGMa has beneficial effects on the PD pathology in 1-methyl-4-phenyl-1,2,3,6-tetrahydropyridine (MPTP)-injected mice. Antibodies against RGMa ameliorate the decrease in dopaminergic neurons, as well as the microglia/macrophage activation induced by MPTP. We assessed the therapeutic effects of intravenous treatment with antibodies against RGMa in the PD model. Finally, we examined the possibility that microglia/macrophage activation could be involved in the decrease of the dopaminergic neuron and motor deficits induced by an increased expression of RGMa in the SN. Our findings suggest that increased RGMa expression in the SN affects the nigrostriatal pathway and inhibition of RGMa with neutralizing antibodies provides therapeutic benefits in PD.

## Results

### MPTP treatment enhances RGMa expression in the SN

**Fig. 1:**
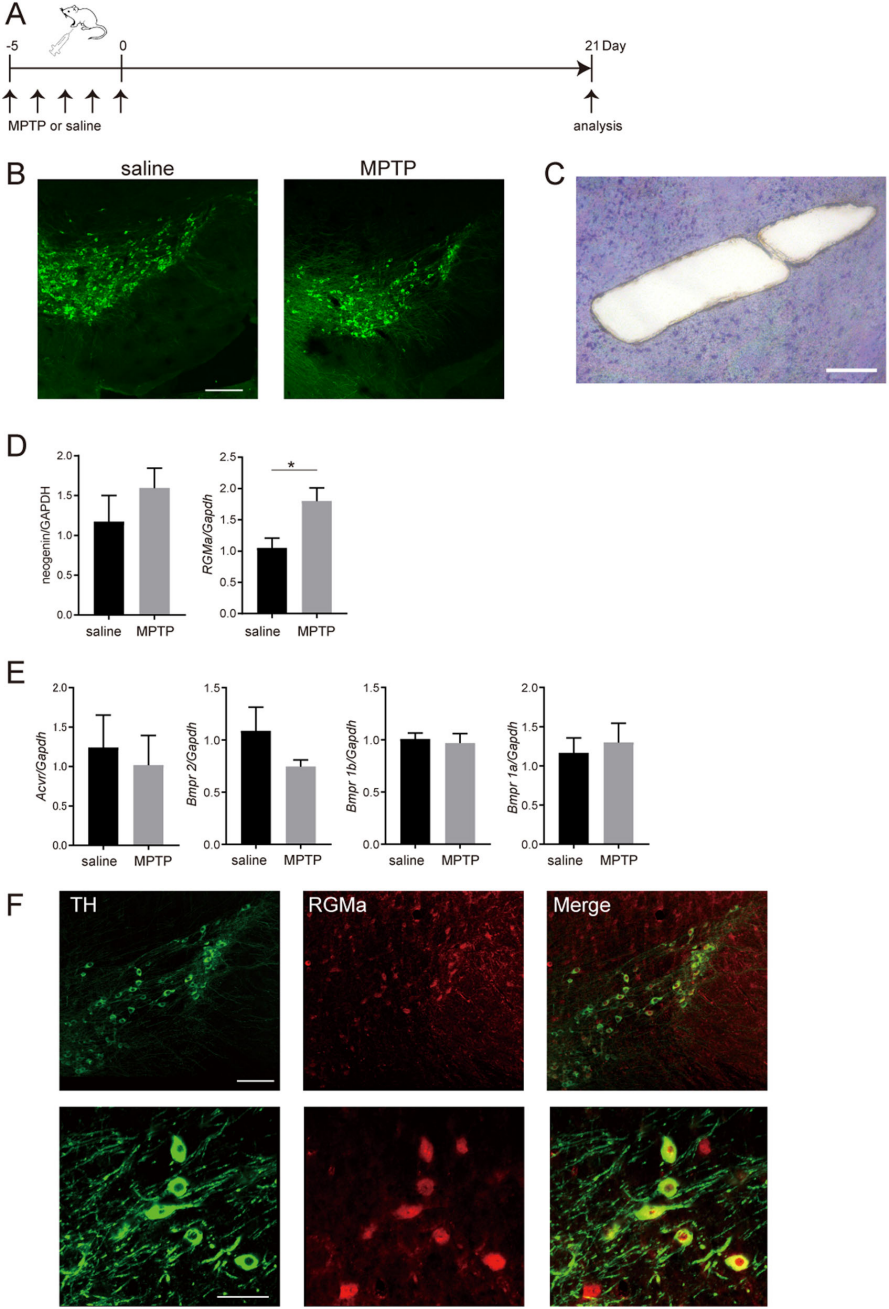
Increased RGMa expression is induced by MPTP injection. **A** Schematic representation of the MPTP injection and sample preparation timeline. **B** Immunofluorescent staining for TH in the SNc of mice injected with saline or MPTP. Scale bar, 200 μm. **C** A representative image after the collection of the SN using laser microdissection. Scale bar, 200 μm. **D**, **E** Relative mRNA expression levels of *RGMa* and *Neogenin* (**D**), and co-receptors for RGMa (**E**) in the SN. The *RGMa* mRNA level is increased in the MPTP-treated group compared with the saline-injected group. *n* = 5. *P < 0.05, Welch’s *t* test. **F** Coimmunostaining of RGMa (red) with the dopaminergic neuronal marker TH (green) in the SN of an MPTP-treated mouse at day 21 after the last MPTP injection. Scale bar, 150 μm.

We administered to mice with the MPTP widely used to develop experimental models of PD^16–21^. The brains were removed after perfusion-fixation 21 days post MPTP injection (Fig. 1A). Sections of substantia nigra were used for immunohistochemistry. MPTP treatment reduced TH-positive neurons in the SN (Fig. 1B). RGMa expression in SN was significantly increased by MPTP injection (*P* = 0.023, *P* < 0.05), however, its receptor neogenin remained unchanged (Fig. 1C, D). While RGMa modulates bone morphogenetic protein (Bmp) signaling^22^, we did not observe significant differences in Bmp receptors including Bmpr1a, Bmpr1b, Bmpr2, and activin receptor (Acvr) (Fig. 1E). RGMa expression was observed in TH-positive dopaminergic neurons in MPTP-treated mice (Fig. 1F).

### Inhibition of RGMa reduces the decrease in TH-positive neurons in a PD mouse model

**Fig. 2:**
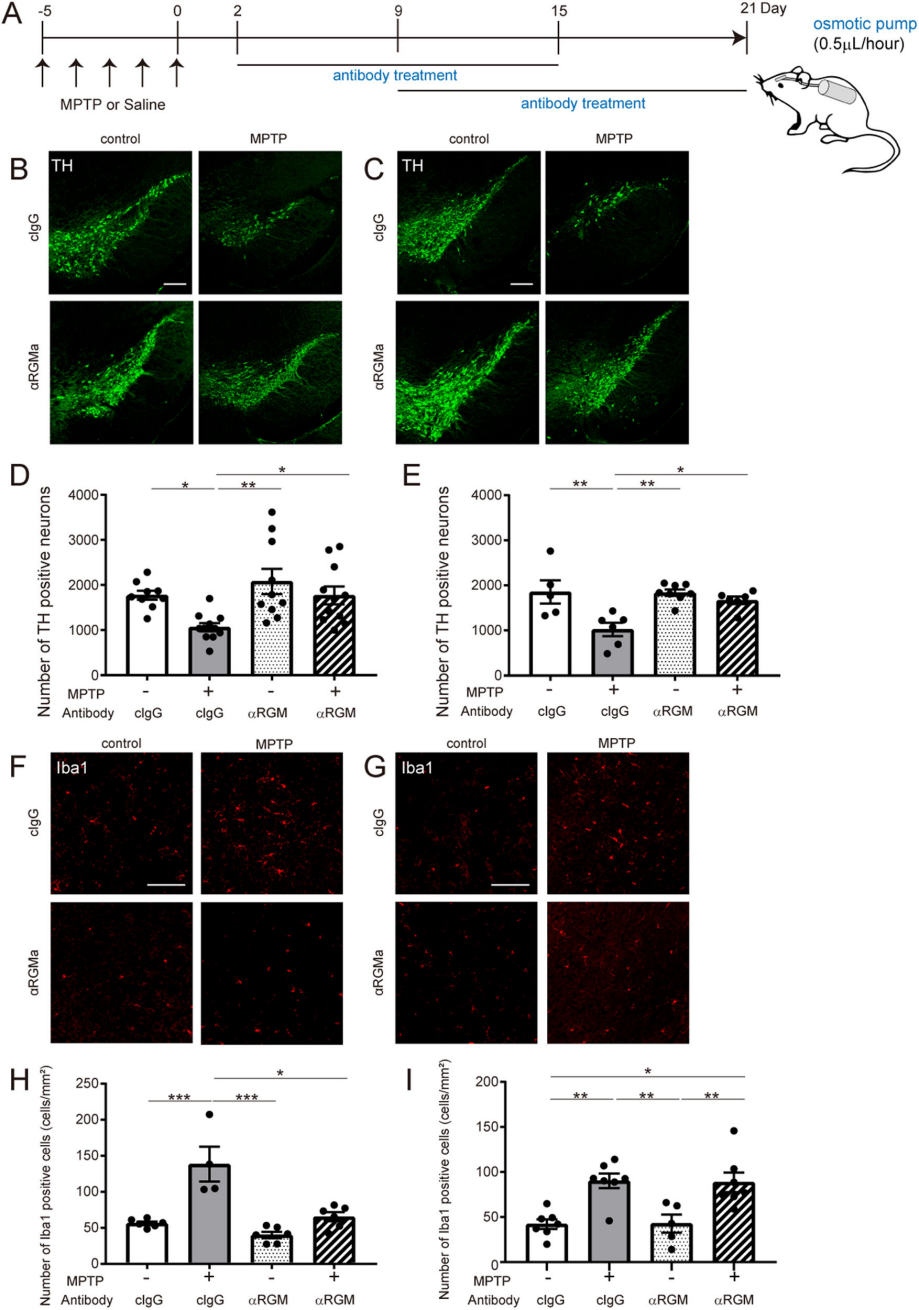
Neutralizing antibodies for RGMa suppress MPTP-induced neuronal loss and inflammation. **A** Schematic representation of the MPTP injection and antibody treatment timeline. **B**, **C** Immunofluorescent staining for TH (green) in the SN of mice injected with saline or MPTP with anti-RGMa antibody or control IgG (cIgG) treatment from day 2 to day 15 (**B**) or from day 9 to day 21 (**C**) after the last MPTP injection. Scale bar, 200 μm. **D**, **E** Stereology counts of TH-positive neurons in the SN of mice treated with anti-RGMa antibody or control IgG from day 2 to day 15 or from day 9 to day 21 after the last MPTP injection. *n* = 5–8 (**F**), *n* = 9–11 (**G**). *P < 0.05, **P < 0.01, two-way ANOVA with Bonferroni multiple comparison test. **F**, **G** Immunofluorescent staining for Iba1 in the SN of mice injected with saline or MPTP with anti-RGMa antibody or control IgG treatment from day 2 to day 15 (**F**) or day 9 to day 21 (**G**) after the last MPTP injection. Scale bar, 100 μm. **H**, **I** Stereology counts of Iba1-positive neurons in the SN of mice treated with anti-RGMa antibody or control IgG from day 2 to day 15 or from day 9 to day 21 after the last MPTP injection. *n* = 4–5 (**H**), *n* = 4–6 (**I**). *P < 0.05, **P < 0.01, ***P < 0.005, two-way ANOVA with Bonferroni multiple comparison test.

To investigate whether RGMa is involved in the PD pathology, we administered an anti-RGMa antibody intraventricularly using osmotic pumps for 2 weeks beginning at 2 days or 9 days after MPTP treatment (Fig. 2A). We found that RGMa inhibition in both phases suppressed the reduction of TH-positive cells (Fig. 2B–E). MPTP induced the accumulation of Iba1-positive microglia/macrophages around the SN (Fig. 2F, G). Both early and delayed treatment of antibody reduced Iba1-positive cells compared to the unspecific IgG treatment as control (Fig. 2F–I). These results suggest that RGMa inhibition ameliorates neuronal damage and inflammation in the MPTP model.

### Intravenous treatment with humanized anti-RGMa antibodies suppresses neuronal loss and inflammation in the PD mouse model

**Fig. 3:**
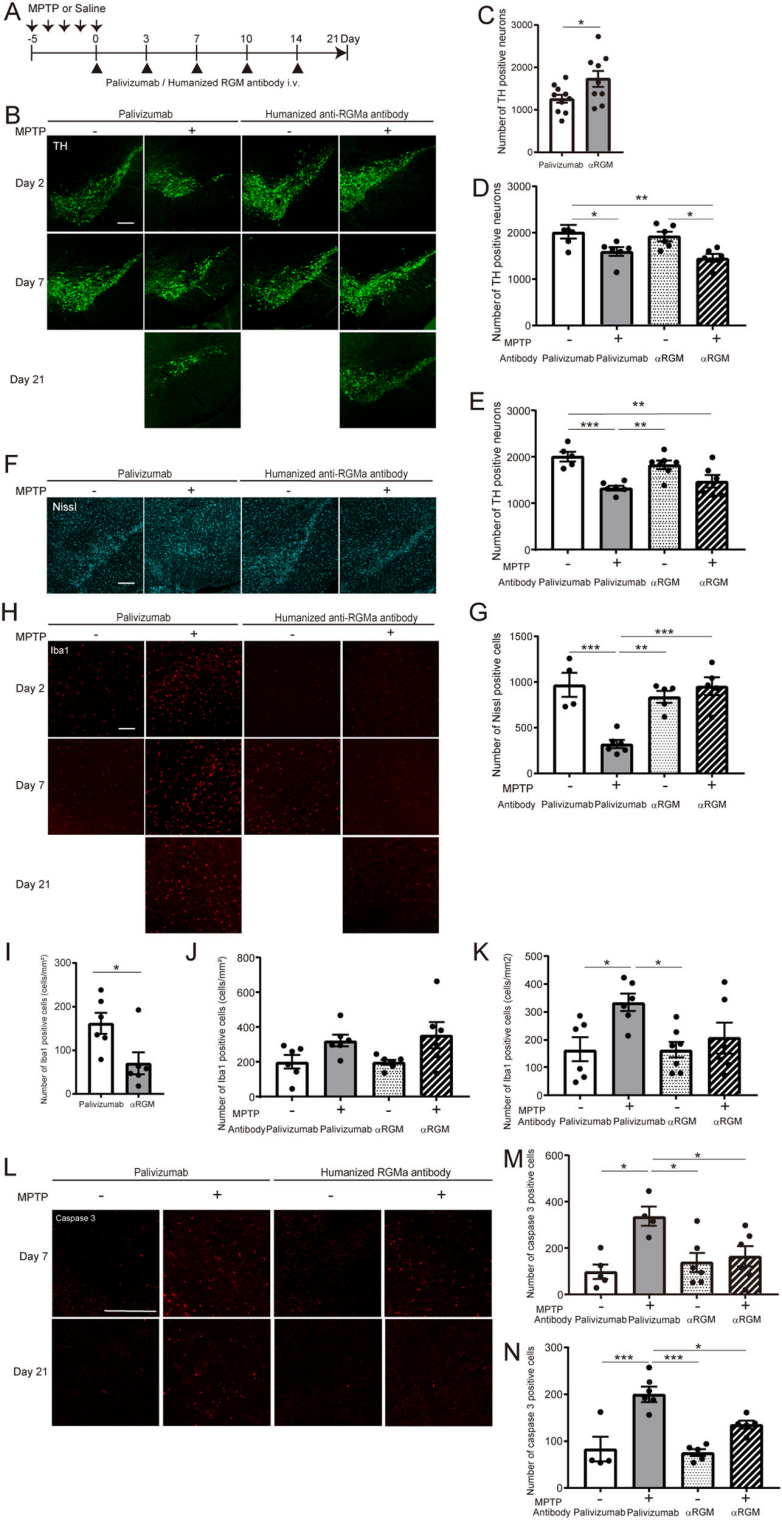
Humanized anti-RGMa antibodies suppress MPTP-induced neuronal loss and inflammation. **A** Schematic representation of the timeline of MPTP injection and humanized antibody treatment. **B** Immunofluorescent staining for TH in the SN at 2 days (top), 7 days (middle), and 21 days (bottom) after the last MPTP injection. Scale bar, 200 μm. **C** Changes in the numbers of TH-positive cells in the SN of MPTP-injected mice with anti-RGMa antibody or Palivizumab (as control) treatment at 21 days after the last MPTP injection. *n* = 9–10. *P < 0.05, Welch’s *t* test. **D**, **E** Stereology counts of TH-positive neurons in the SN from mice with anti-RGMa antibody or control (Palivizumab) treatment at 2 days (**D**) and 7 days (**E**) after the last MPTP injection. *n* = 5–6 (**D**), *n* = 5–7 (**E**). *P < 0.05, **P < 0.01, ***P < 0.005, two-way ANOVA with Bonferroni multiple comparison test. **F** Immunofluorescent staining for Nissl in the SN at 21 days after the last MPTP injection. Scale bar, 200 μm. **G** Stereology counts of Nissl-positive neurons in the SN from mice with anti-RGMa antibody or control (Palivizumab) treatment at 21 after the last MPTP injection. *n* = 4–6. **P < 0.01, ***P < 0.005, two-way ANOVA with Bonferroni multiple comparison test. **H** Immunofluorescent staining for Iba1 in the SN at 2 days (top), 7 days (middle), and 21 days (bottom) after the last MPTP injection. Scale bar, 100 μm. **I** Changes in the numbers of Iba1-positive cells in the SN of MPTP-injected mice with anti-RGMa antibody or Palivizumab treatment at 21 days after the last MPTP injection. *n* = 5–6. *P < 0.05, Student’s *t* test. **J**, **K** Stereology counts of Iba1-positive neurons in the SN of mice with anti-RGMa antibody or control (Palivizumab) treatment at 2 days (**J**) and 7 days (**K**) after the last MPTP injection. *n* = 5–6 (**J**), *n* = 5–7 (**K**). *P < 0.05, one-way ANOVA with Bonferroni multiple comparison test. **L** Immunofluorescent staining for cleaved caspase3 in the SN at 7 days (top) and 21 days (bottom) after the last MPTP injection. Scale bar, 50 μm. **M**, **N** Stereology counts of cleaved caspase3-positive cells in the SN of mice with anti-RGMa antibody or control (Palivizumab) treatment at 7 days (**M**) and 21 days (**N**) after the last MPTP injection. *n* = 4–6 (M), *n* = 4–5 (**N**). *P < 0.05, **P < 0.01, ***P < 0.005, one-way ANOVA with Bonferroni multiple comparison test.

A humanized monoclonal anti-human RGMa antibody caused therapeutic effects on axonal degeneration, inflammation, and functional recovery from motor deficit on the model of MS^14,23^. We intravenously injected this antibody at 0, 3, 7, 10, and 14 days after MPTP treatment (Fig. 3A). Palivizumab was used as an isotype control for the anti-RGMa antibody. Anti-RGMa antibody suppressed the decrease in TH-positive neurons by MPTP injection at 21 days post-injection (Fig. 3B–E). Nissl-positive neurons also decreased 21 days after MPTP injection and anti-RGMa antibody suppressed this decrease (Fig. 3F, G). Iba1-positive microglia/macrophages increased at 21 days after MPTP injection, and this increase was significantly reduced by the intravenous treatment with the humanized anti-RGMa antibody (Fig. 3H–K). Cleaved caspase3-positive apoptotic cells significantly increased after MPTP treatment at 7 and 21 days after MPTP treatment (Fig. 3L–N). Anti-RGMa antibody suppressed this increase caused by MPTP treatment (Fig. 3M, N). These results suggest that humanized anti-RGMa antibodies attenuate neuropathological damages and inflammatory responses in the animal model of PD.

### AAV-mediated RGMa overexpression in the SN causes neuronal degeneration and motor impairment

**Fig. 4:**
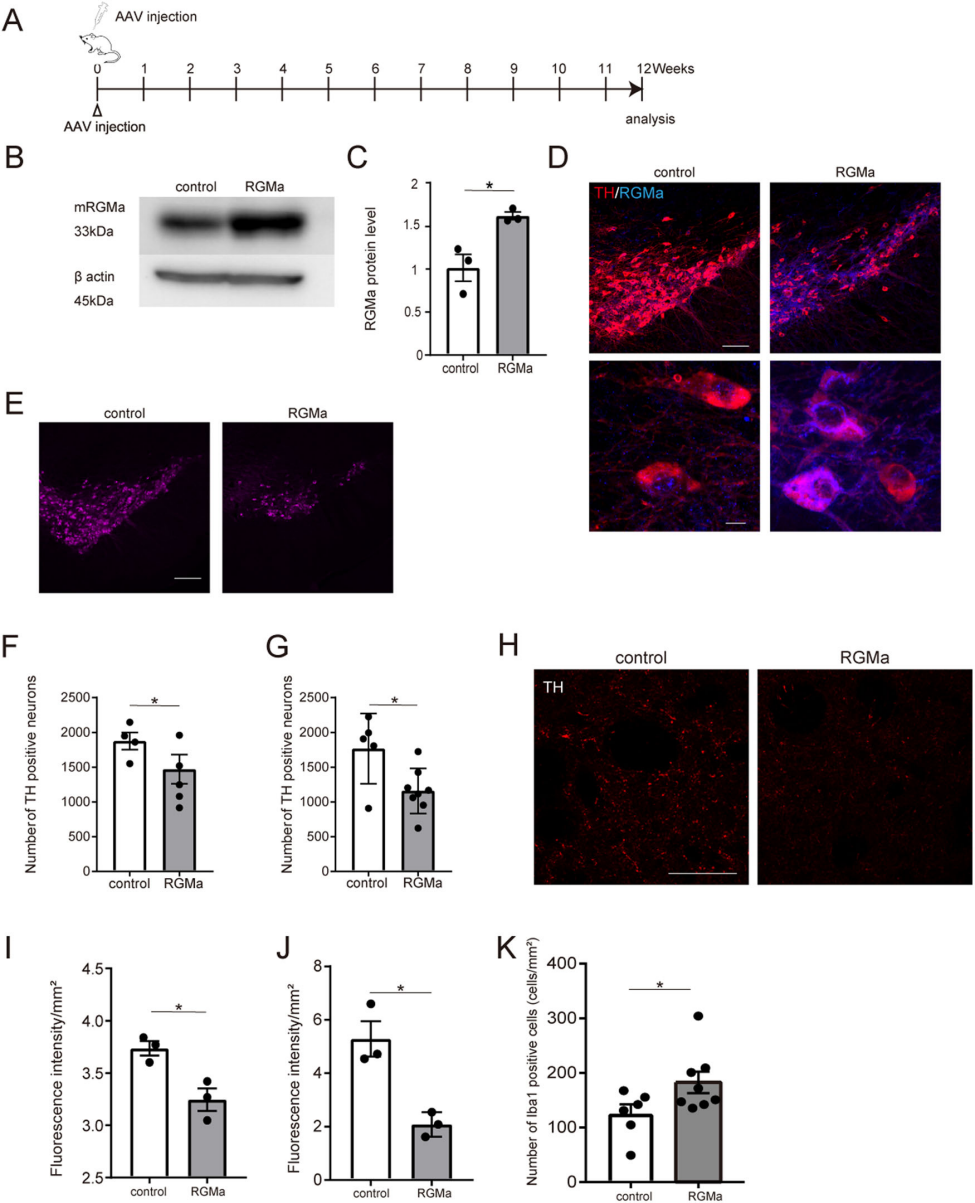
AAV-mediated RGMa overexpression induces neuronal degeneration in the nigrostriatal pathway. **A** Schematic representation of the timeline of AAV injection in the SN. RGMa-encoding AAV was injected into the bilateral SN. GFP-encoding AAV was used as a control. **B** Western blot analysis showing the increased expression of RGMa protein in the SN. Beta-actin was used as the loading control. **C** Quantitative data for **B**. RGMa protein expression was significantly higher in mice injected with RGMa-encoding AAV compared to GFP-injected control animals. *n* = 3. *P < 0.05, Student’s *t* test. **D** Coimmunostaining for RGMa and TH in the SN of mice injected 12 weeks after AAV injection. Low magnification (top): Scale bar, 200 μm. High magnification (bottom); scale bar, 10 μm. **E** Immunofluorescent staining for TH in the SN of mice injected 12 weeks after AAV injection. Scale bar, 200 μm. **F**, **G** Stereology counts of TH-positive neurons in the SN of mice 8 weeks (**F**) and 12 weeks (**G**) after AAV injection. *n* = 4–5 (**F**), *n* = 5–8 (**G**). *P < 0.05, Student’s *t* test. **H** Immunofluorescent staining for TH in the striatum of mice injected 12 weeks after AAV injection. Scale bar, 50 μm. **I**, **J** Relative TH fluorescence intensities were measured in the striatum at 8 weeks (**I**) and 12 weeks (**J**) after AAV injection using the ImageJ software. *n* = 3, each group. *P < 0.05, Student’s *t* test. **K** Stereology counts of Iba1-positive neurons in the SN of mice 12 weeks after AAV injection. *n* = 6–9. *P < 0.05, Student’s *t* test.

To investigate the effects of RGMa on the PD pathogenesis, we injected adeno-associated virus (AAV) carrying the RGMa gene under the TH promoter into the SN (Fig. 4A). RGMa protein levels increased 4 weeks after AAV injection (Fig. 4B, C). RGMa signal increased in SN of mice 12 weeks after AAV injection (Fig. 4D). Histological analysis revealed that RGMa overexpression significantly reduced TH-positive neurons in the SN 8 weeks (Fig. 4E, F) and 12 weeks after AAV injection (Fig. 4G). TH-positive neuronal fibers in the striatum decreased from 8 to 12 weeks after AAV injection by RGMa overexpression (Fig. 4H–J). RGMa overexpression induced the accumulation of microglia/macrophages around SN (Fig. 4K).

**Fig. 5:**
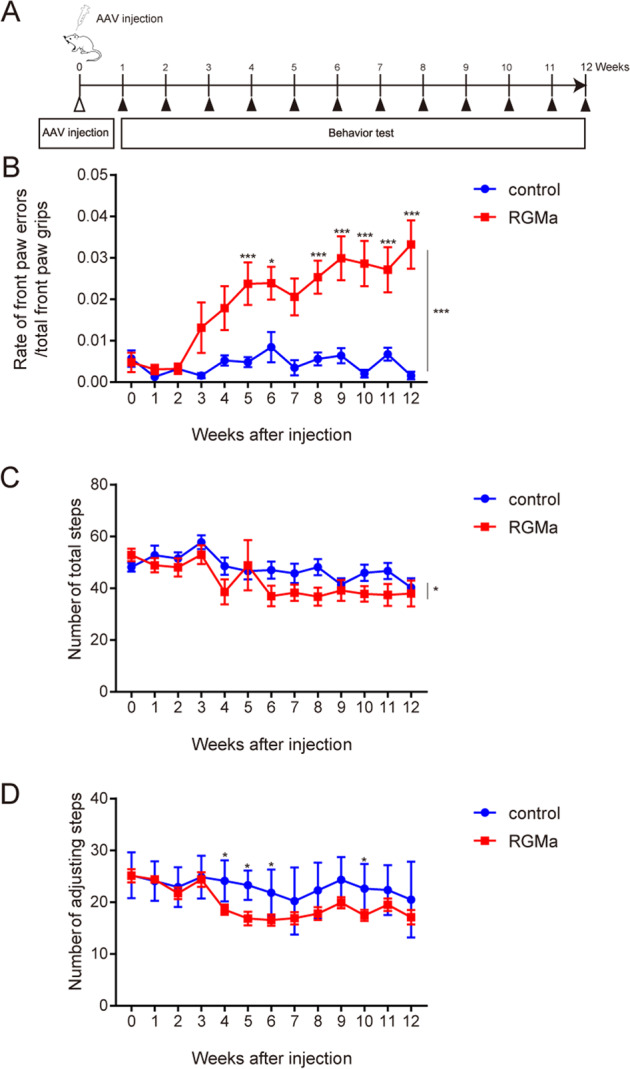
Increased RGMa in the SN induces motor impairments. **A** Schematic representation of the timeline of AAV injection in the SN and the behavioral tests. **B**, **C** Time-dependent changes in forepaw faults (**B**) and the number of total steps (**C**) in the grid test were measured in mice injected with AAV. *n* = 17–18. *P < 0.05, **P < 0.01, ***P < 0.005, two-way repeated-measures ANOVA with Bonferroni multiple comparison test. **D** Increased expression of RGMa causes a decrease in adjusting steps as measured by the stepping test. *n* = 17–18. *P < 0.05, two-way repeated-measures ANOVA with Bonferroni multiple comparison test.

To assess the effects of RGMa overexpression on behavior, we performed behavioral tests assessing motor impairments caused by the decline of dopamine in the striatum (Fig. 5A)^24,25^. RGMa overexpression induced front paw errors compared to saline-treated controls from 5 weeks after AAV injection in Grid test (*P* = 0.02, *P* < 0.01) (Fig. 5B). The number of total steps in the Grid test did not decrease, thus animal activity was immutable (Fig. 5C). In the stepping test, RGMa overexpression decreased the numbers of adjusting steps at 4 weeks after AAV injection (*P* = 0.007, *P* < 0.01), but this disfunction came back to control levels at the end of the experiment (Fig. 5D). This change of adjusting steps was small and transient. RGMa overexpression is involved in the degeneration and loss of TH-positive neurons, as well as in the neuronal deficits in the PD.

### RGMa causes morphological activations of microglia

**Fig. 6:**
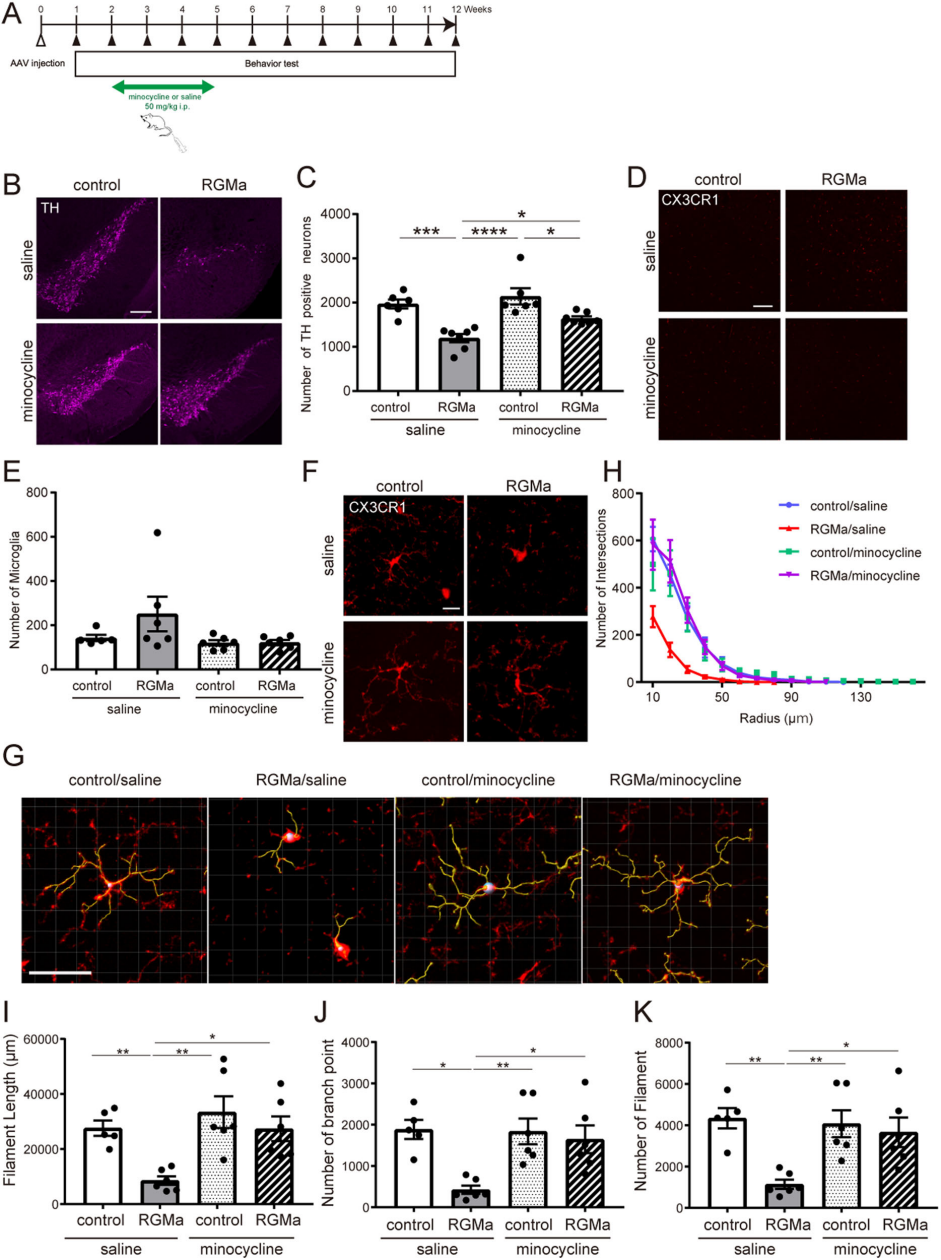
RGMa activates microglia morphologically. **A** Schematic representation of the timeline of AAV injection, intraperitoneal minocycline administration, and behavioral tests. The white arrowhead indicates the time point of AAV injection; the black arrowhead indicates the time point of the behavioral tests. **B** Representative images of the immunofluorescent staining for TH in the SN at 5 weeks after AAV injection. Scale bar, 200 μm. **C** Changes in the numbers of TH-positive cells in the SN of AAV-injected mice with saline or minocycline treatment at 5 weeks after AAV injection. *n* = 5–6. *P < 0.05, one-way ANOVA with Bonferroni multiple comparison test. **D** Representative images of microglia in the SN of Cx3xr1CreERT2; Ai14 mice 5 weeks after AAV injection with or without minocycline treatment. Scale bar, 100 μm. **E** Changes in the numbers of CX3CR1-positive microglia in the SN of AAV-injected mice with saline or minocycline treatment at 5 weeks after AAV injection. *n* = 6. **F** Representative images of microglial morphology in the SN of Cx3xr1CreERT2; Ai14 mice 5 weeks after AAV injection with or without minocycline treatment. Scale bar, 10 μm. **G** Tracing image of microglia by Imaris. Microglia changed to an activated form by overexpression of RGMa 5 weeks after AAV injection. Scale bar, 30 μm. **H** Results of sholl analysis. Microglia of mice overexpressed RGMa had shortened processes near the cell body. Minocycline treatment improved the number and length of processes. *n* = 5–6. **I**–**K** Measured values of microglial morphology. In AAV-TH-RGMa treated group, the length of microglial processes (**I**), the numbers of branch point (**J**), and processes (**K**) were significantly reduced, and administration of minocycline suppressed morphological changes. *n* = 5–6. *P < 0.05, **P < 0.01, one-way ANOVA with Bonferroni multiple comparison test.

Microglial activation is involved in the inhibitory effect of RGMa on axonal growth^26^, we thus investigated whether activation of microglia/macrophages was responsible for the pathology of the increased RGMa expression in the SN. Minocycline inhibits glutamate-induced activation of p38 MAPK of microglia^26^. Minocycline suppresses an activation, proliferation, and inflammatory response of microglia caused by neuronal damage^27^. Thus we used minocycline for inhibits microglia/macrophage activation in this model (Fig. 6A). RGMa overexpression in the SN decreased TH-positive cells (Fig. 6B, C). Minocycline suppressed the degeneration of TH-positive neurons induced by RGMa overexpression (Fig. 6C).

Microglia were specifically labeled using the tamoxifen-inducible Cre-LoxP system^28^. The chemokine (C-X3-C motif) receptor 1 (CX3CR1) is expressed mainly in microglia, but it is also expressed in peripheral mononuclear phagocytes under inflammatory conditions^28^. Cx3cr1CreERT2 mice carrying the fluorescence reporter induce Cre recombination in these cells, leading to fluorescence labeling after tamoxifen treatment. Since microglia showed delayed clearance compared to other peripheral mononuclear phagocytes, most fluorescence-labeled cells remaining 4 weeks after tamoxifen treatment are microglia^28^. Cx3cr1CreERT2; Ai14 mice were treated with tamoxifen to specifically label microglia with tdTomato fluorescence. Minocycline treatment did not cause an accumulation of microglia caused by RGMa overexpression around SN (Fig. 6D, E). However, overexpression of RGMa shortened microglial processes (Fig. 6G, I) and reduced processes and branches at 5 weeks after AAV treatment (Fig. 6H, J, K). RGMa overexpression induced morphological activations of microglia and minocycline treatment suppressed this morphological change. Minocycline administration suppressed the morphological changes of microglia caused RGMa overexpression. These results suggest that increased RGMa in the SN activates microglia.

### RGMa overexpression in the SN causes motor impairments by a microglia/macrophage-dependent mechanism

**Fig. 7:**
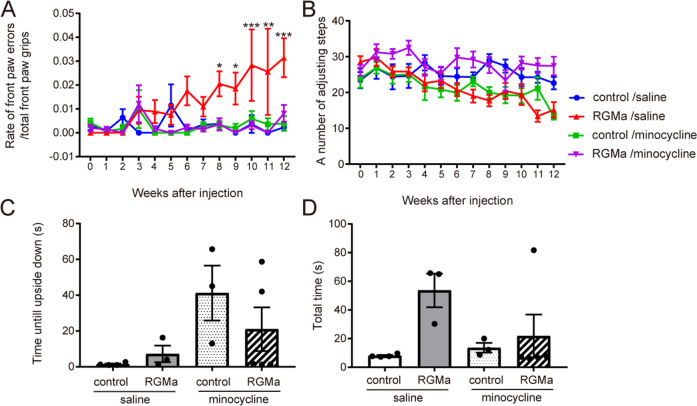
Minocycline treatment ameliorates RGMa-induced motor deficits. **A** Time-dependent changes in forepaw faults during the grid test were measured in mice injected with AAV after administration of minocycline. Minocycline suppresses motor impairments induced by increases in RGMa levels. *n* = 6–9. *P < 0.05, **P < 0.01, ***P < 0.005, two-way repeated-measures ANOVA with Bonferroni multiple comparison test. **B** Time-dependent changes in adjusting steps during the stepping test were measured in mice injected with AAV after administration of minocycline. *n* = 6–9. No significant difference in the two-way repeated-measures ANOVA with Bonferroni multiple comparison test. **C** Time-dependent changes in average time to upside down were measured in the pole test in minocycline-treated mice. *n* = 3–4. One-way ANOVA with Bonferroni multiple comparison test. **D** The total time to descend to the floor was measured in the pole test after minocycline treatment. *n* = 3–4. One-way ANOVA with Bonferroni multiple comparison test.

Minocycline treatment alleviated the increased front paw errors in the grid walk test in RGMa-overexpressing mice (Fig. 7A). There is no significant effect of RGMa overexpression on the stepping test and pole test (Fig. 7B–D). These results suggest that the activation of microglia is partially involved in motor deficits caused by RGMa.

### RGMa increases the mRNA expression of *Tnfα* in microglia

**Fig. 8:**
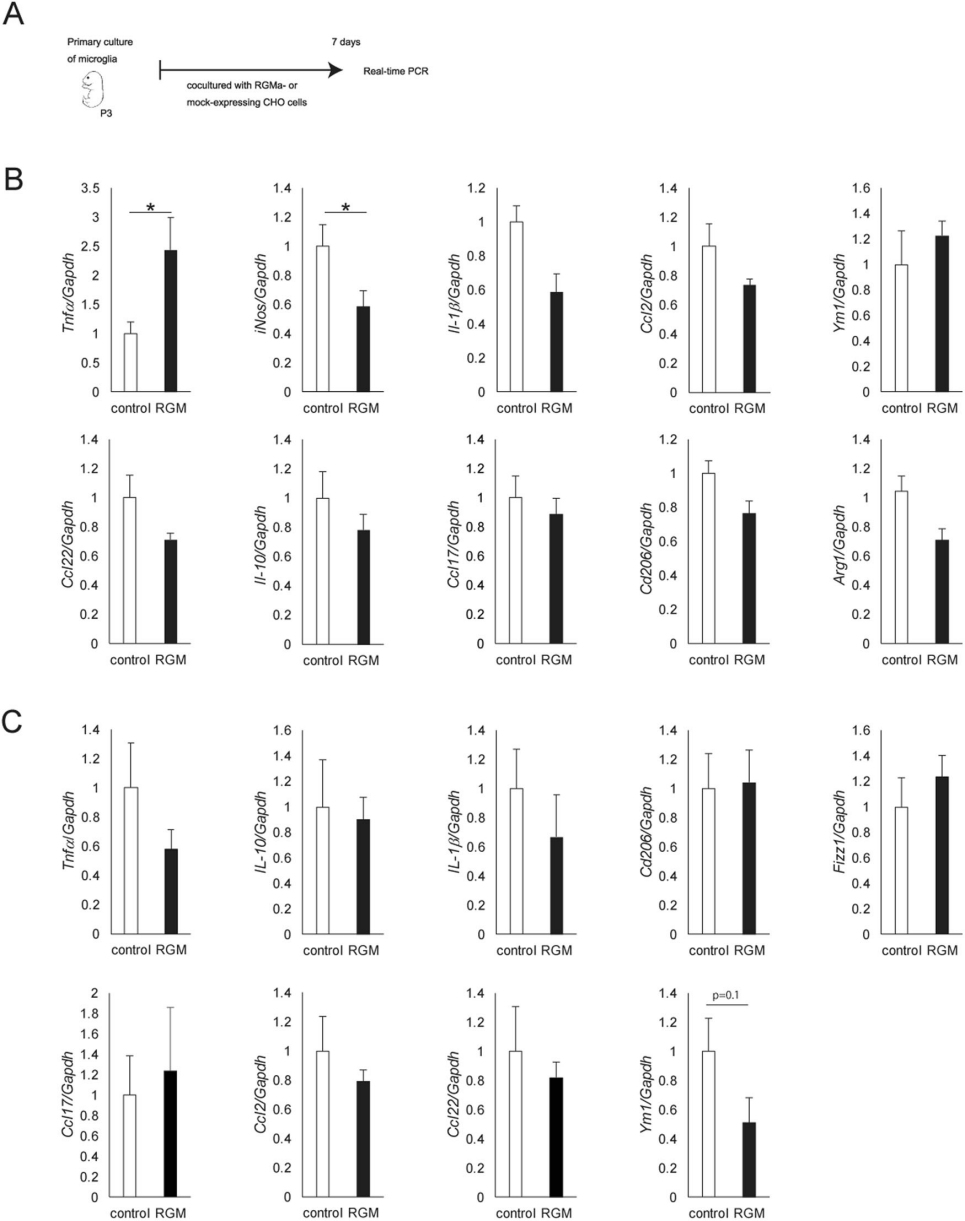
RGMa increases mRNA expression of *Tnfα* in microglia. **A** Schematic representation of the timeline of primary culture, and treatment with RGMa. **B**, **C** Relative mRNA expression of pro-inflammatory cytokines, chemokines, and M1 or M2 markers in cultured microglia (**B**) and in microglia isolated from brains of Cx3cr1CreERT2; Ai14 mice (**C**). The mRNA level of *Tnfα* is increased in the microglia cocultured with RGMa compared with control. *n* = 4–5 (**B**), *n* = 3–4 (**C**). *P < 0.05, Student’s *t* test.

Accumulation of activated microglia in the SN has been found in PD^29,30^. Activated microglia release various inflammatory cytokines and elicit an abnormal inflammatory response in PD^31,32^. To evaluate the direct effects of RGMa to microglia without the involvement of other cells, we cocultured microglia collected from embryonic mice cortex and RGM expressing CHO cells (Fig. 8A). RGMa upregulated the genes related to pro-inflammatory pathways such as tumor necrosis factor (*Tnfα*) in cocultured microglia with RGMa-CHO cells, suggesting that RGMa activates microglia directly (Fig. 8B) (*P* = 0.030, *P* < 0.05). We treated Cx3cr1CreERT2; Ai14 mice with tamoxifen and collected microglia using fluorescence-activated cell sorting. RGMa could not reduce the expression of genes associated with tolerance of inflammation such as Ym1 (Fig. 8C) (*P* = 0.10). These results support that increased RGMa in the SN induces microglial activation directly.

## Discussion

We demonstrated the therapeutic effects of anti-RGMa antibodies on the pathology in an animal model of PD. Both the intraventricular treatment with polyclonal anti-RGMa antibodies and the intravenous treatment with humanized monoclonal anti-RGMa antibodies suppressed the decrease in the number of dopaminergic neurons and the accumulation of microglia/macrophages induced by MPTP administration. Increased expression of RGMa in the SN led to the degeneration of the nigrostriatal pathway and caused the activation of microglia/macrophages, which may result in the observed motor deficits. Minocycline ameliorated the RGMa-induced deleterious effects. The aberrant activation of the immune system might be involved in exacerbations of RGMa-induced neuropathological changes and motor deficits.

Two-week intrathecal administration of polyclonal anti-RGMa antibodies promotes axon growth of the corticospinal tract and motor recovery following SCI^14^. Our results demonstrated that continuous intraventricular administration for 2 weeks with the anti-RGMa antibody, which reached the cerebrospinal fluid, had sufficient therapeutic effects. A delayed start of the treatment until 9 days after MPTP injection also attenuated the degeneration of dopaminergic neurons in the SN of MPTP-injected mice; this allows for a wider therapeutic time window.

An intravenous bolus administration offers some advantages compared with a continuous intrathecal administration including prevention of infections via the administration route and lower maintenance costs. We demonstrated that not only intraventricular injection of anti-RGMa antibodies but also the intravenous injection of humanized anti-RGMa antibodies showed beneficial effects in the PD model. These data implicate that targeting RGMa with antibodies would be a useful strategy for the treatment of PD.

The neurodegeneration of the dopaminergic pathway is an important aspect of PD. Our data demonstrate that virus-mediated RGMa overexpression under the TH promoter caused the degeneration of dopaminergic neurons in the SN and motor dysfunction. These findings are consistent with a previous study that injected AAV encoding RGMa under the synapsin1 promoter^15^. Motor dysfunction caused by RGMa overexpression was partial, and some function came back to control levels at the end of the experiment. TH level in surviving neurons increases as a compensation for the neuronal death in MPTP model mice, virus-mediated RGMa overexpression mice, and synucleinopathy model mice^15,33,34^. This compensation for the neuronal death could recover motor function. Downregulation of Rho kinase and ROCK, which are activated downstream of RGMa and mediates axonal growth inhibition, protects dopaminergic neurons in animal models of PD^35,36^. Neuronal RGMa expression might be involved in the loss of dopaminergic neurons in PD.

Inflammation is another prominent pathological feature in PD^37–43^. The reduced microglial activation in the MPTP-injected mice supports the survival of dopaminergic neurons^44^. We observed that anti-RGMa antibodies reduced the microglia/macrophage activation after MPTP injection. Minocycline, which inhibits the activation of microglia, ameliorated RGMa-induced neuronal loss/degeneration and motor deficits in this model. In vitro experiments using cultured microglia were performed to reveal the direct effects of RGMa on microglia. Cultured microglia revealed that RGMa upregulated the expression of *Tnfα*, a gene associated with inflammation in microglia. Inhibition of RGMa is implicated not only in neurodegeneration but also in inflammation in animal models including SCI, MS, and neuromyelitis optica^10,13,14,24^.

The mechanisms by which increased RGMa levels in the SN shift microglia/macrophages into a pro-inflammatory activated state remain unknown. RGMa expressed in microglia affects axonal growth via Neogenin in neurons^26^. The motor impairments caused by RGMa overexpression thought to result from degeneration of dopaminergic neurons and phagocytosis of microglia activated by degenerated nerves. It is presumed that the inflammatory response due to activated microglia in the SN is not seen in the early stage of PD but seen in an advanced stage. We need to consider a detailed time course. There are two intriguing possibilities of how TH-neuron-specific RGMa overexpression regulates microglial activation, in brief, RGMa-induced neuropathological hallmarks of PD. (1) Increased RGMa expression in neurons directly activates microglia/macrophage and caused neuronal degeneration by releasing inflammatory cytokines. (2) Microglia are activated in response to a degeneration of dopaminergic neurons. The progression of neuronal damages in the SN is induced on both pathways. Cell type-specific knockdown of genes encoding neuropathic cytokines that causes neuronal degeneration would be helpful to elucidate the molecular mechanisms for the RGMa-mediated activation of microglia/macrophages in the SN.

Since RGMa has various physiological functions and expressed in multiple cell types, anti-RGMa antibodies exert various therapeutic mechanisms. RGMa-neogenin signaling is involved in the pathogenesis of diverse neurological disorders and inhibition of RGMa exert beneficial effects^10,13,24,45–47^. Particularly, the humanized monoclonal anti-RGMa antibody demonstrated therapeutic effects on axonal degeneration, inflammation, demyelination, and behavioral recovery in animal models of MS^15^. Our results suggest that RGMa would likely be involved in neurodegeneration and inflammation; antibody-based RGMa inhibition on the immune system and the CNS might contribute to the treatment of PD. Inflammation and neurodegeneration are common denominators among diverse neurodegenerative diseases. Our findings provide the possibility that the inhibition of neurodegeneration and inflammation using humanized monoclonal anti-RGMa antibodies could be a common therapeutic approach for neurodegenerative diseases.

## Materials and methods

### Animals

Adult male C57BL/6J mice (8 weeks of age; Japan SLC Inc.) were used in this study. Cx3cr1CreERT2 mice^28^ and Ai14 (B6. Cg-Gt(ROSA)26Sor^tm14(CAG-tdTomato)Hze^/J) mice^48^ were obtained from The Jackson Laboratory (Bar Harbor). All experimental procedures were approved by the Institutional Animal Care Committee of Osaka University and complied with the guidelines for the care and use of laboratory animals of Osaka University.

### MPTP administration

The mice received a single intraperitoneal injection of MPTP (30 mg/kg; MedChem Express) daily for 5 consecutive days. Control mice received saline administrations following the same time and dosage schedules^49^.

### Laser microdissection

Procedures were performed according to the previous study^50^. Fresh-frozen brains of 21 days after MPTP injection were used. The tissues were continuously cut into 10-μm-thick coronal sections using a cryostat. SN was dissected bilaterally with an LMD 7000 (Leica Microsystems).

### Quantitative polymerase chain reaction

We analyzed gene expression of SN tissues in MPTP-treated mice, RGMa overexpressed mice, and in cultured microglia. The procedures were performed according to the previous study^50^. The relative expression was normalized to *Gapdh*. The primer sequences are described in Supplementary Table S1.

### Immunohistochemistry

Procedures were performed according to the previous study^50^. The tissues were cut into 20-µm-thick coronal sections using a cryostat. We used the M.O.M kit (Vector Laboratories) for immunohistochemistry following the manufacturer’s protocol. The sections were incubated with primary antibody overnight at 4 °C. Anti TH antibody (1:1000; MAB 5280, Millipore,), rabbit anti-Iba1 (1:1000; 019-19741, Wako), rabbit anti-cleaved caspase3 (1:800; Cell Signaling Technology), and rabbit anti-RGMa (1:1000; MAB2458, R&D Systems) were used. The sections were incubated with the following secondary antibodies for 1 h at RT. Alexa Fluor 488-, 647-conjugated streptavidin (1:1000; Invitrogen), Goat-anti-rabbit IgG Alexa 568 or 647 (1:1000; Invitrogen) were used. To assess the non-TH-dependent neuronal loss, we stained Nissl (NeuroTrace^TM^ Fluorescent Nissl Stains, 1:200, Molecular Probes, Inc.) for 1 h at room temperature. Images were acquired with a confocal laser-scanning microscope (FV1200, FV3000; Olympus).

### Stereology counts of the dopaminergic neuron, microglia, and cell death

The loss of neurons in the SN was determined by serial section analysis of the total number of TH-positive neurons, as previously described^51^. Every fourth coronal section of 20-μm thickness throughout the entire rostral-caudal axis of the mesencephalon was analyzed. The total TH^+^ neuron populations in SN on both sides were calculated. The activation of microglia and cell death in the SN was determined by serial section analysis of Iba1 and caspase3.

Six coronal sections every 200 μm of 20-μm thickness in SN on both sides were analyzed.

### Striatal densitometry

Quantifications of striatal dopaminergic fibrous staining were performed on striatal tissues from mice 8 and 12 weeks after AAV injection^15^. The anatomical borders of the striatum were defined using the mouse brain atlas^52^. The area of cortex just outside the striatum was outlined to obtain a measurement of the background fluorescence levels using the ImageJ (National Institutes of Health). Total fluorescence intensities of TH were measured in six sections per animal.

### Antibody treatment

Osmotic minipumps (Durect Co.) were implanted into the right lateral ventricle 1.0 mm rostral and 2.2 mm to the right of the bregma with a cannula extending to a depth of 2.5 mm from the skull surface^53^. Pumps filled with control rabbit IgG (22.3 μg/kg/day; Sigma-Aldrich) or anti-RGMa antibody (28045 Immuno-Biological Laboratories) were surgically implanted 2 or 9 days post MPTP injection^10^. We injected humanized anti-RGMa antibody or Palivizumab (developed by Mitsubishi-Tanabe Pharma Co.), which neutralizes the effects of RGMa, intravenously (10 mg/kg) after MPTP injection on day 0 and then days 3, 7, 10, and 14.

### Behavioral tests

#### Grid test

The grid test was used to study forepaw use, in particular, the use of the distal musculature and digit manipulation, which is sensitive to dopaminergic input from the striatum. Mice were suspended upside down on a metal grid and allowed to move freely across the grid for 30 s. The average ratio between the total forepaw faults/total forepaw steps over these trials was calculated^24,54^.

#### Stepping test

Mice were allowed to settle at one edge of an open table (1 m in length). The experimenter lifted the hind legs by pulling up the tail and pulled mice backward by the tail at a steady speed of approximately. Each animal performed three trials and the average number of adjusting steps from both forepaws was calculated^25^.

#### Pole test

The pole test was performed with slight modifications^55,56^. Briefly, mice were placed on the top of a vertical wooden pole (diameter: 1 cm; height: 55 cm) with their heads up. The total time to descend to the floor and the time until they headed down was recorded. If the mouse descended partly and finally slipped down from the pole, a default value of 30 s was recorded. If the mouse was unable to descend but slipped down from the pole, a default value of 60 s was recorded.

### AAV production

AAV purification was performed as previously described^57^. pAAV2.5-THP-GFP/WGA was a gift from Kwang-Soo Kim (Addgene plasmid # 80337)^58^. Full-length RGMa was amplified from pAAV-RGMa and subcloned into the pAAV2.5-THP-GFP/WGA vector instead of GFP. pAAV2.5-THP-GFP/WGA or pAAV2.5-THP-RGMa/WGA, Rep/Cap plasmid, as well as helper plasmid, were transfected into cultured 293 AAV cells. Five days after transfection, cells were collected and rAAV was purified. Titration was performed using the AAVpro Titration Kit (6233, TaKaRa Bio). Genomic titers were 3.4 × 10^9^ gc/µl (GFP), 4.0 × 10^9^ gc/µl (RGMa). We injected 2 μl of these AAV into mice brain.

### AAV vector validation by western blot analysis

The SN was isolated from mice 12 weeks after AAV injection using a dissecting microscope. Tissues were lysed in lysis buffer containing 5 M NaCl, 10% NP40, 1 M Tris-HCl (pH 7.4), 50% glycerol, and cOmplete protease inhibitor cocktail (Roche Applied Science). Each sample was heated in 6× loading buffer at 95 °C for 5 min, resolved by sodium dodecyl sulfate-polyacrylamide gel electrophoresis, and transferred to polyvinylidene fluoride membranes (Millipore). Membranes were treated with blocking solution (1% milk in 1× phosphate-buffered saline with 0.1% Tween 20^®^) for 30 min and incubated with rabbit anti-RGMa (1:100, 28045, Immuno-Biological Laboratories) and rabbit anti-β-actin (1:1000, 4970; Cell Signaling Technology) overnight at 4 °C. The membranes were incubated with horseradish peroxidase-conjugated secondary antibody against rabbit IgG (1:1000; Cell Signaling Technology) for 1 h in RT. Detection was performed using western blotting Substrate Plus (Thermo Fisher Scientific).

### Surgical procedures

The AAV injection procedures were slightly modified from a previous study^15^. Mice were deeply anesthetized, and 1 μl/site of AAV were injected using glass capillaries connected to a microsyringe (Ito). The injection coordinates from bregma were −2.8 mm anteroposterior, ±1.3 mm lateral, and −4.3 mm ventrodorsal from the dura.

### Sorting of microglia

Microglial cells were prepared from Cx3cr1CreERT2; Ai14 mice^28,59,60^. Mice treated with tamoxifen and AAV were anesthetized 5 weeks after AAV injection. Cerebral cortexes were removed from the brain. Tissues were mechanically dissociated by the Miltenyi GentleMACS tissue dissociator using the Adult Brain Dissociation Kit (Miltenyi) following the manufacturer’s protocol. We used mouse CD11b-biotin antibody (130-113-804; Miltenyi) and Anti-biotin MicroBeads ultrapure (130-105-637; Miltenyi) to purify CD11b-positive cells. Then tdTomato-expressing cells in collected populations were sorted on a FACSAria III (BD). The cells were subjected to RNA extraction and quantitative real-time PCR.

### Culture of microglia with RGMa-expressing cells

Microglial cells were obtained from C57BL/6J mice on P6 for the primary culture^61,62^. Briefly, the cerebral cortex was digested with 0.25% trypsin and DNase for 15 min at 37 °C. Cells were passed through 70-µm nylon mesh. The resultant cell suspension was diluted with DMEM supplemented with fetal bovine serum (FBS; 10%) and penicillin (100 U/ml)/streptomycin (100 µg/ml) and plated onto poly-L-lysine-coated dishes. After 12 days, microglial cells detached from astrocyte monolayer sheets were collected by shaking and plated on the upper filter surface of transwell plates (pore size, 3 µm; Corning). These microglial cells were cultured with RGMa-expressing CHO cells plated in a transwell plate for 7 days and subjected to RNA extraction for qPCR.

### Statistics

Results are presented as the mean ± SEM. Statistical analyses were performed using GraphPad Prism 7 (GraphPad). Results were analyzed using Student’s *t* tests, Welch’s *t* test, one-way ANOVA, and two-way ANOVA followed by Bonferroni tests. All *P* values <0.05 were considered statistically significant.

## Supplementary information

Supplementary figure legend

Supplementary Table 1
