# Supplementary material for: Inhibition of repulsive guidance molecule-a protects dopaminergic neurons in a mouse model of Parkinson’s disease
Source: Cell Death Dis. 2021 Feb 15;12(2):181. doi: 10.1038/s41419-021-03469-2 (PMC7884441; doi:10.1038/s41419-021-03469-2)
Supplement: Supplementary file 1 — Supplementary figure legend [file 41419_2021_3469_MOESM1_ESM.docx]

**Supplemental Figure Legends**

Supplementary Table 1. Primers for quantitative real-time PCR.
